# Participation of Children with Spina Bifida: A Scoping Review Using the International Classification of Functioning, Disability and Health for Children and Youth (ICF-CY) as a Reference Framework

**DOI:** 10.3390/medicina54030040

**Published:** 2018-05-30

**Authors:** Indrė Bakanienė, Laura Žiukienė, Vaida Vasiliauskienė, Audronė Prasauskienė

**Affiliations:** Department of Children’s Rehabilitation, Lithuanian University of Health Sciences, LT-47179 Kaunas, Lithuania; laura.bigelyte@gmail.com (L.Ž.); vasiliauskiene.vaida@gmail.com (V.V.); a.prasauskiene@gmail.com (A.P.)

**Keywords:** children, environment, involvement, participation, spina bifida

## Abstract

*Background and objectives.* Participation is a fundamental right of the child, regardless of his health status. Assessing and supporting the participation of children with spina bifida (SB) presents a significant challenge for practitioners. The purpose of this study was to examine what is known about the participation of children with SB. *Materials and Methods.* The framework for scoping reviews from Arksey & O’Malley was used. A literature search in Cumulative Index to Nursing and Allied Health Literature (CINAHL), Medical Literature Analysis and Retrieval System Online (Medline), PsychINFO and the Education Resources Information Centre (ERIC) databases retrieved 136 papers, 10 of which met the criteria for inclusion and were selected for analysis. Synthesis of the results on participation within occupational domains of leisure, school and community, and factors influencing participation of children with SB was performed. *Results.* All the included studies were non-experimental and used cross-sectional, population-based or qualitative design. Most studies analysed social participation or participation in physical activities, except one that focused on school participation. Data from these studies have shown that children with SB experience greater participation restrictions compared to their typical peers or children with other chronic diseases. The participation was mainly affected by contextual factors. Relationships between pathology and participation were not sufficiently validated. *Conclusions.* There is little research on the participation of children with SB. Future studies must consider contextual factors and interventions facilitating or impeding participation.

## 1. Introduction

The World Health Organization (2001) outlines participation as “an involvement in all life situations” that include domestic life, school [1], and recreational activities that are all considered to be a key outcome of health [2]. The participation component includes two aspects of the individual’s experience: attendance and involvement. Attendance is defined as the frequency of attending and diversity of activities, while involvement is defined as the more subjective experience of participation related to motivation, persistence, social connection, and affect [3].

The International Classification of Functioning, Disability and Health (ICF) model identifies participation as a complex phenomenon that changes over time and is affected by the interaction of body structures and functions with environmental and personal factors [1]. Children’s participation is crucial because it directly affects their behavioural and emotional well-being, social relationships, and mental and physical health [4]. Compared to children without disabilities, children with disabilities tend to participate in fewer school, recreational, and social activities, and the diversity of their participation declines as they get older [5].

Spina bifida (SB) is the most prevalent neural tube defect and occurs early after conception when the neural tube does not close properly. This defect results in a malformation of the spinal cord and very often in malformations of the brain. These structural changes lead to paralysis and loss of sensation below the affected level, incontinence, and cognitive impairment. Secondary impairments include deformities of the legs, feet, and back, endocrine dysfunction, pressure sores, and pain [6].

SB is the most complex congenital anomaly compatible with long-term survival [7], with up to 70–80% of children born with SB surviving into adulthood [7,8]. The increased life expectancy has made researchers and practitioners turn to the promotion of the optimal well-being, functioning, and participation across all lifespan, rather than just ensuring the survival of the child. Even though the participation of children with SB is becoming a more common subject, there have not been any review papers conducted to date. This paper aims to systematically review the research done in this field and determine future research needs.

## 2. Materials and Methods

A scoping review method was the most suitable to explore papers related to the research question. It aims to examine the literature in an area of interest concerning the extent, nature, and peculiarities of the primary research [9]. Research commonly uses a scoping review method when the topic has not yet been comprehensively investigated or reviewed or is of a complex or heterogeneous nature [10].

For this review, the methodology described by Arksey and O’Malley (2005) was used. This methodology divides the review into the following main phases: identifying the research question, search for relevant studies, and selection of studies, charting the data, collating, summarising, and reporting the results [9].

The research question was: “What are the scientific knowledge about the participation of children and youth with SB?”

During February 2018, the databases for CINAHL, Medline, PsychINFO, and ERIC were searched for articles published from 1990 onwards. The search strategy used the formula: children AND spina bifida AND participation. Search terms for “children” included “pediatric”, “children”, “adolescence”, ”adolescents”, “youth”, “young adults”, “young people”. Search terms for spina bifida included “myelomeningocele”, “spina bifida”, and “spinal dysraphism”. Search terms for participation involved terms “participation”, “involvement”, “engagement”, “leisure participation”, “school participation”, “community engagement”, and “social participation”. Subject headings were defined and adjusted for each database. Also, the references of relevant reviews and all included papers were examined for eligible articles missed during the initial electronic searches. An Internet search (scholar.google.com) using the terms “spina bifida”, “myelomeningocele”, “children”, and “participation” was performed to find any relevant articles or grey literature. The language was restricted to English.

Two reviewers (IB and LZ) independently screened the titles and abstracts of the selected studies according to the specific inclusion criteria. Disagreements about inclusion were discussed and a consensus was achieved. The eligibility criteria were: (1) articles in English; (2) participants: children aged 0–18 with SB; (3) research that exclusively studied participation (e.g., attendance or involvement) of children with SB in daily activities. Studies examining two populations (SB and one other) and comparing participation issues between these populations were also included. No restrictions were applied related to the type of design of studies identified during the primary search. For all selected abstracts the full text was downloaded and analysed by IB and re-evaluated by LZ. In the cases where the opinions of IB and LZ have differed regarding the appropriateness of the article, a third person (VV) was included to reach a final decision.

Two reviewers independently identified a set of variables that could be used to describe the studies. A data-charting form was developed. It contained: (1) general information about the study (author(s), year of publication, study location); (2) specific information related to the study population, aims of the research, study design, participation measures, participation data, associations between any medical, socio-demographic, psychological, and intervention-related determinants and participation. Then IB and LZ independently extracted the data and interpreted the results.

Data collation and summarisation was performed in two steps. A descriptive summary of included studies was prepared, which included the following information: authors, year, and country, the purpose of the research, study design, sample, and primary results (Table 1). A narrative synthesis was performed to outline data on participation. Results were arranged in four areas according to the ICF model: (1) participation within occupational domains of leisure, school, and community; (2) body structure and function; (3) personal factors; (4) environment.

## 3. Results

**Figure 1:**
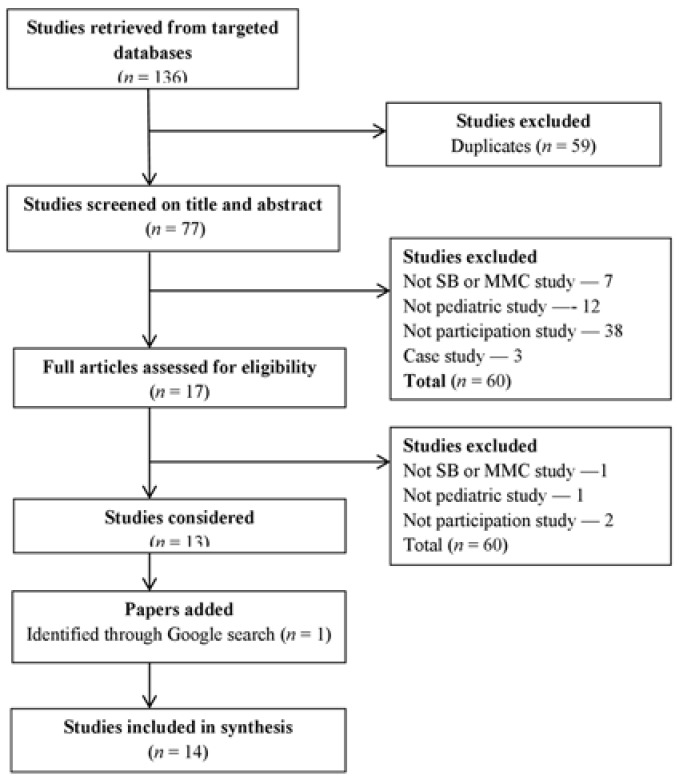
Flowchart of our mapping process and study selection. SB, spina bifida; MMC, myelomeningocele.

In total, 77 articles were identified in CINAHL, Medline, PsychINFO, and ERIC after duplicates (*n* = 59) were removed. In addition to these, an internet browser search yielded 18 publications. After screening the titles, abstracts, and full texts, 10 studies appeared to be relevant to the research question (Figure 1).

Half of the studies were conducted in the United States, while others were from the Netherlands, Canada, Sweden, and Australia (Table 1). Of the 10 articles selected, eight specifically explored the participation of children with SB. Two studies compared participation issues between SB and acquired spinal cord injury (ASCI) (1 study) and cystic fibrosis (CF) (1 study).

All the research was non-experimental: seven studies used cross-sectional, one population-based, and two qualitative study designs. Most studies analysed social participation or participation in physical activities and recreation, and one study focused exclusively on school participation. Participation was measured using four validated and five non-validated tools. Reliability and validity was established for all standardised tests: Children’s Assessment of Participation and Enjoyment (CAPE) [11], Availability and Participation Scale (APS) [12], School function assessment (SFA) [13], and Assessment of Preschool Children’s Participation (APCP) [14]. Characteristics and results of all included studies are presented in Table 1. Instrument data are found in Table 2.

### 3.1. Social Participation

Social participation was analysed in five studies: one research paper used qualitative methodology [22] while others were large quantitative studies exploring different aspects of community participation [18,20,21,22]. Three cross-sectional studies were focused on attendance issues [20,21,22] and found that children with SB had lower social participation rates than their healthy peers [20,22] and children with other disorders (learning disabilities or asthma) [21]. The study conducted by Flanagan et al. (2013) compared different dimensions of attendance and involvement in social activities for children with early-onset ASCI and SB. The results of the study indicated that both groups had similar levels of participation intensity and enjoyment. However, participants with SB were involved in fewer activities when compared to those with paraplegia due to ASCI. Surprisingly, participation of the SB group was more similar to the ASCI group with tetraplegia than ASCI group with paraplegia [18].

The methodologically robust qualitative study investigated the experiences of parents and children with SB related to social participation and peer relationships. Most parents who participated in interviews did not report their children as “typical” and pointed out a lot of differences between their child with SB and other children in the domains of peer relationships and social participation. Children with SB rated the effect of SB on their daily functioning from minimal to significant social isolation and rejection [16]. The paper does not specify which aspect of participation (e.g., attendance or involvement) was related to “being different”.

### 3.2. Participating in Sports and Recreational Activities

A frequency of participation (i.e., attendance) in physical activities of children with SB was analysed in four quantitative cross-sectional studies [17,20,22,24]. The extensive research of Connor-Kuntz et al. (1995) that used non validated questionnaire reported a high level of participation in sports for children with SB [24]. Three more recent studies [17,20,22] showed conflicting data. In the studies conducted by Boudos et al. (2008) and Marques et al. (2015), less than 40% of participants were involved in both organised and non-organised physical activities [17,22]. The study of Boudos and Mukherjee (2008) revealed that 20% of children participated in physical activities more than once a week, 5% once a week, and 12% at least once a month [22].

### 3.3. Participating in School

Attendance and involvement in school participation have been examined by one population-based, cross-sectional study from Sweden. The results of this study showed that children with SB participated a lot in school activities, mostly in structured activities. However, involvement in those activities was quite low especially in the setting of recess/playground [19]. Another study by Connor-Kuntz et al. (1995) investigated the frequency of participation (i.e., attendance) in physical education. The authors pointed out that most children with SB received physical education. However, more than one-third of the sample had no opportunity to participate in regular physical education with their healthy peers during the previous school year upon which reporting was based [24].

### 3.4. Factors Influencing Participation

The studies have found a variety of positive and negative elements related to the body structure and functions, person, and environment that may reduce or facilitate participation.

#### 3.4.1. Body Function and Structures

Four studies reported bowel and bladder problems (mainly when the children were unable to catheterise themselves) as barriers to physical activities [15] and social life [16,20,22]. The experience reported by the children and parents in the qualitative study of Fischer et al. (2015) ranged from the minimal effect of incontinence on daily activities to social isolation and rejection. The ability to self-catheterisation and continence were named as important facilitators of community participation and peer relationships [16].

The relation between participation and other medical issues (cognitive dysfunction, hydrocephalus, obesity, and pain) were analysed in several papers. Cognitive abilities had a positive impact on physical participation in the study of Bloemen at al. (2015) [15]. Kelly et al. (2011) have found that youth without a shunt and recent major medical issues participated in a wider range of physical and skill-based activities [20]. The qualitative research of Bloemen et al. (2015) indicated that injuries, pain, orthopaedic deformities, and obesity were important negative factors affecting participation in sport and recreational activities [15].

#### 3.4.2. Activity Limitations

Relationships between motor functioning and participation issues were analysed in three studies with different results [17,20,24]. One study did not reveal any relationship between community participation and level of lesion and walking abilities [20]. In contrast, the data from other study showed that motor skills were the most significant determinant of the engagement in the classroom and mealtime/snack time activities [19]. Moreover, Connor-Kuntz et al. (1995) determined a contrasting association whereby children with independence in ambulation reported lower sports participation. Children who use wheelchairs participated in a larger number of formal sports activities than those who walk using an assistive device [24]. Competence in both simple and complex abilities (transfers, wheelchair skills) was the important positive factor for physical activities [15]. Issues with communication and learning disability had adverse effects on community participation in the study of Liptak et al. (2010) [21]. Peny-Dahlstrand et al. (2013) indicated that processing skills were the important contributory factor in the setting of a classroom, mealtime/snacks, and playground/recess [19].

#### 3.4.3. Personal Factors

The most important positive factors that contribute to participation were self-confidence, positive experience, a solution-oriented approach [15], motivation/desire for participation [15,22], a perception of competence [17]. Barriers included lack of motivation [22], mood/fear [15], language barriers [15,22]. Older children participated less in recreational, physical, and skill-based activities [20].

#### 3.4.4. Environment

Children’s everyday environments had a strong impact on participation. Barriers to participation were revealed in school and work environments, physical and built environment, within institutional and government policies, services and assistance, attitudes and social support. The most significant barriers to participation were: poverty [21,22], lack of family support and time [22], insufficient information [15,22,23], lack of transportation and community programmes, unsafe environment [22], shortage and limited access to playgrounds and sport facilities, the overprotective attitude towards children and adolescents with disabilities, the inability of parents and/or teachers to be open-minded and flexible [15]. Facilitators for participation included caregivers employment [20], information for parents, a solution-oriented approach within the family, parental encouragement of physically active and independent lifestyles for their children [15], good assistive devices for mobility and personal care [15,24], and services/support at school [21].

## 4. Discussion

Information about the participation of children with SB was collected from 10 studies and classified under the guidance of the ICF framework. Most studies analysed social participation or participation in recreational and physical activities, except one that focused on school participation. Findings from these studies demonstrated that children with SB had lower participation rates and involvement than their typical peers or children with other chronic diseases. Participation was mainly affected by personal factors and the environment, while relationships between pathology and participation were not sufficiently validated.

A significant strength of this review is the systematic and broad search that likely captured all of the relevant papers. Attendance and involvement issues, as well as factors related to participation in every domain of the ICF, were systematically summarised, which enables us to see all available evidence and reveal areas for further research. However, the study is not without limitations: a quality appraisal of selected studies was not performed. Also, the studies reviewed have used a variety of different designs and showcased different levels of quality.

Participation is considered to be the most important outcome of rehabilitation interventions [25] and a primary human right [26]. There is a great need for parents, service providers, and policymakers to understand the nature, patterns, and amounts of children’s participation in order to provide successful interventions and improve overall well-being. Despite the importance of the research implications, participation of children with SB is insufficient is insufficiently investigated. Out of the few studies conducted regarding the participation of children with SB, the majority displayed significant limitations. While the intensity dimension (e.g., attendance) of participation has been explored in almost all of the papers, the dimensions related to involvement have often been neglected. This is an issue because when researchers only measure the intensity of participation, the children who take part in a lot of activities but have little involvement will be assigned to those who have high levels of participation. Another problem with this methodological approach is that it fails to represent the diversity of the activities, as to receive the intensity score it is enough to take part in only one type of activity [27]. Furthermore, only a few studies used valid and reliable instruments to measure participation.

The majority of studies reviewed were focused on community participation, and these studies were the most elaborate ones due to a higher number of children and measures with adequately demonstrated reliability and validity. Studies exploring participation in school, domestic life, and leisure were fewer in number, creating a great need for large methodologically strong population-based studies investigating different aspects of the participation of children with SB across all life situations.

The findings of the scoping review showed that children with SB tend to participate in fewer activities and at a reduced intensity than typically developing children or children with other types of physical disabilities. For example, Law et al. (2006) investigated the participation of 427 children with different types of physical disabilities and found sufficiently high participation in social activities and particular in free activities [28].

Although children with SB and paraplegic ASCI have many physical similarities, the frequency of attending of children with SB was lower than that of children with paraplegic ASCI. The results showed that the participation of children with SB was the same as that of children with ASCI who had tetraplegia [18]. These findings could be explained by the differences related to the age at onset (congenital vs acquired) and cognitive issues. Children born with disabilities are seen as being more vulnerable by their parents compared to their healthy peers. Such attitude can lead to a situation of overprotection where the child with SB is no longer aware of his/her capabilities. Holmbeck et al. (2002, 2003) noted that parents of children with SB were much more overprotective than parents of children without a medical condition. The overprotected children did not have enough decision-making autonomy, social maturity, or interactions outside the school, and were dependent on adults for guidance [29,30]. Children with acquired disabilities more successfully avoid overprotection and its consequences, especially if the injury occurs at an older age. Other differences between children with ASCI and SB are the high prevalence of hydrocephalus and its complications, and cognitive dysfunction in children with SB [31]. Individuals with SB often have difficulties with executive functioning and memory, which result in a disordered ability to initiate, plan, solve problems, and act on their initiative [31,32]. Research has proven that the most critical and prevalent barrier to participation is low motivation, which may be influenced by several factors such as a lack of experience, limited peer interactions, neurological impairment and “learned helplessness”. Children with disabilities who have never experienced high levels of participation and peer interactions may not know an alternative way and may be less encouraged to participate in social activities [24,31]. Furthermore, neurological impairment and overprotection by the parents can result in reduced goal-directed and initiation behaviours [32]. These findings suggest that the interventions should be targeted towards increasing motivation levels based on each child’s interests. It is recommended that children be subjected to interventions at a younger age before negative environmental influences have appeared and a behavioural pattern has been established.

The present scoping review proposes that all environmental domains in the ICF had a direct influence on the participation of children with SB. However, those findings are not specific to children with SB; the participation of children with all types of chronic diseases is greatly influenced by environmental barriers [15,19].

The review shows that there is currently insufficient evidence related to body structures and functions (e.g., level of lesion, ambulation, hydrocephalus, incontinence) and participation in children with SB, which can be explained by the conceptual model proposed by King et al. (2003) [33]. Children’s health condition and functional issues had a direct effect on participation, as well as an indirect effect through the environment. The direct effects are mediated by environmental factors, meaning that without their presence, the impact of health conditions on participation would have been observed [25,33]. In order to improve function and participation, researchers and practitioners should direct their attention toward the environment and other context-based therapies, rather than the child’s impairments.

This paper points out the considerable lack of interventions aimed at promoting participation and suggests strategies to reduce the barriers to participation. Interventions tailored to enhance participation in all areas of life should be the primary focus of future research.

## 5. Conclusions

Studies describing the participation of children with SB are limited. Most research has been focused on community participation, and many predictive studies only examined child-related factors. The majority of quantitative studies were cross-sectional, so longitudinal and intervention studies that focus on participation in all life situations at a biological, occupational, and environmental level are needed.

## Figures and Tables

**Table 1:** Summary of the research that examined the participation of children with spina bifida.

First Author (Year), Country	Sample N (N By Groups), Age	Design: Focus	Participation/Main Outcome Measure	Main Findings Related to Participation
Bloemen et al. (2015) [15], The Netherlands	Spina bifida (SB) *N* = 33 (8–18) (children); *N* = 42 (parent)	Qualitative, grounded theory: factors affecting physical activity	Physical activity for persons with disability (PAD) model: intention, attitude, self-efficacy, health condition, facilitators and barriers, intention, social influence.	Personal factors related to participation were: bowel and bladder care, competence in skills, sufficient fitness, medical events, and self-efficacy. Environmental ones were: support from other people, assistive devices for mobility and care, information related to possibilities for adapted sports and accessibility of sports facilities.
Fischer et al. (2015) [16], Canada	SB *N* = 11 (children) *N* = 10 (parents)	Qualitative, phenomenology: the experiences around continence issues, social participation, and peer relationships	Semi-structured interview (1) normal versus different; (2) independence, ownership and the road to continence; (3) peer relationships and acceptance	Children with SB that achieved bladder continence were more independent and participated in more social activities.
Marques et. al. (2015) [17], Portugal	SB *N* = 31 (10–17)	Quantitative, cross-sectional: psychosocial correlates of physical activity	Physical activity and psychosocial survey	Only 38.7% of the children participated in both organised and non-organised physical activity. Results did not support the relationship between participation in physical activities and psychosocial correlates. Only perception of competence (OR = 9.55, 1.06–85.99, *p* < 0.05) had the positive association with participation in non-organized physical activity
Flanagan et al. (2013) [18], USA	*N* = 137 (5–18) SB *n* = 54 Spinal cord injury (SCI) = 83	Quantitative, cross-sectional: psychosocial outcomes (including community participation) of children and adolescents with early-onset SCI and SB	Children’s Assessment of Participation and Enjoyment (CAPE)	Children with SCI and SB had similar levels of participation with intensity score 2.1 for children with SB and 2.2 for children with SCI (out of a possible 7). Participants with SB participated in fewer activities (diversity score 23.9 out of possible 55) than those with paraplegic SCI (diversity score 23.9)
Peny-Dahlstrand et al. (2013) [19], Sweden	SB *N* = 50 (6–14)	Quantitative, population-based, cross-sectional cool participation, motor and process skills in task performance	Availability and Participation Scale (APS), School function assessment (SFA)	89.6% of children showed a low level of active participation and involvement in school activities even though their frequency of participation was high. Motor and process skills were the most significant determinant of participation.
Kelly et al. (2011) [20], USA	SB *N* = 63 *n* = 19 (2–5) *n* = 21 (6–12) *n* = 23 (13–18)	Quantitative, cross-sectional: demographic and SB related factors affecting community participation	Assessment of Preschool Children’s Participation (APCP), CAPE	Adolescent’s participation in recreational, physical, and skill-based activities was lower compared to younger children. Social participation was positively associated with caregiver employment. Physical and skill-based activities were negatively related to the presence of a shunt and recent major medical issues. Bladder and bowel incontinence was a barrier to participation for children ages 6–12.
Liptak et al. (2010) [21], USA	SB *N* = 130 (13–17)	Quantitative, population-based, prospective: outcomes and factors affecting social participation	International Classification of Functioning, Disability and Health (ICF) based survey	76% of adolescents with SB were competitively employed or attended school, 15% spend time with friends and were going on dates, and 30% had a driver’s license or learner’s permit.
Boudos et al. (2008) [22], USA	SB *N* = 101 (10–32) *n* = 31 (10–17)	Quantitative, cross-sectional: community participation and barriers to community participation	Medical conditions, function, psychosocial issues, activities, community participation survey	Only 30% of children with SB took part in an organised social activity at least once a week. The most frequent barriers identified were low motivation (38%), lack of information (25%) and time constraints (21%).
Field et al. (2001) [23], Australia	*N* = 166 SB = 97 cystic fibrosis (CF) = 69	Quantitative, cross-sectional: sport and recreational activities	Sport and recreation facilities survey	63% of parents of children with SB and 23% of parents of children with CF reported that their children had limited variety of opportunities for sport and recreation activities.
Connor-Kuntz et al. (1995) [24], USA	SB *N* = 133 (7–16)	Quantitative, cross-sectional: physical education and sport participation	Physical education and sports participation survey	One-third of children with SB did not have the opportunity to participate in physical education with their nondisabled peers. The lowest non-school sports participation was observed for children with SB who walked independently.

Abbreviations: APS, Availability and Participation Scale; APCP, Assessment of Preschool Children’s Participation; CAPE, Children’s Assessment of Participation and Enjoyment; CF—cystic fibrosis; SB, spina bifida; SCI, spinal cord injury; PAD, the physical activity for persons with disability model; SFA, School function assessment.

**Table 2:** Description of the participation measures.

Measurement	Age Range/ Respondent	Purpose	Content	Scale/Items	Reliability	Validity
Assessment of Preschool Children’s Participation (APCP) [14]	2 to 5 years and 11 months (children with/without disabilities) Parent	Participation	Activity in the areas of play, skill development, active physical recreation, and social	Diversity and intensity scores in 5 areas: play, skill development, active physical recreation, social activities, and total 45 drawings of everyday activities	*	**
Availability and Participation Scale (APS) [19]	Elementary/high school (5–18 years (children with disabilities) Teacher	Participation, environment	School activities, school environment	2 scales: availability (27 items), participation (29 items)	*	*
Children’s Assessment of Participation and Enjoyment (CAPE) [18,20]	6–21 years (children with/without disabilities) Self-administered and interviewer-assisted version	Participation	Activity outside mandated school tasks	2 domains: Informal (40 items) formal (15 items) 5 dimensions assessed for each domain: diversity, intensity, with whom, where, enjoyment, preference 5 activity types: recreational, active physical, social, skill-based, self-improvement	**	**
School function assessment (SFA) [19]	Elementary/primary school (5–12 years) School professionals	Activity, participation	School/related functional tasks	3 parts: participation, task support, activity performance	**	***

(a) (*) one type of reliability (internal consistency or test-retest) was tested, with acceptable results; (**) reliability was acceptable in two aspects: internal consistency and test-retest stability >0.70 in 70% or more dimensions. (b) (*) one type of validity (e.g., structural, construct and/or criterion) has been tested, with acceptable results; (**) two types of validity were tested with acceptable results; (***) three types of validity tested with acceptable results.
